# Molecular Evidence for Cryptic Speciation in the *Cyclophorus fulguratus* (Pfeiffer, 1854) Species Complex (Caenogastropoda: Cyclophoridae) with Description of New Species

**DOI:** 10.1371/journal.pone.0109785

**Published:** 2014-10-09

**Authors:** Nattawadee Nantarat, Christopher M. Wade, Ekgachai Jeratthitikul, Chirasak Sutcharit, Somsak Panha

**Affiliations:** 1 Biological Sciences Program, Faculty of Science, Chulalongkorn University, Bangkok, Thailand; 2 Animal Systematics Research Unit, Department of Biology, Faculty of Science, Chulalongkorn University, Bangkok, Thailand; 3 School of Life Sciences, University of Nottingham, University Park, Nottingham, United Kingdom; Tel-Aviv University, Israel

## Abstract

A high degree of intraspecific variation, both genetic and in shell morphology, of the operculate land snail *Cyclophorus fulguratus* (Pfeiffer, 1854) suggests that its classification as a single species warrants reconsideration. We sequenced two nuclear (18S and 28S) and two mitochondrial (16S and COI) genes of 46 *C. fulguratus* specimens and used them to estimate the phylogeny and to determine the validity of species boundaries. Molecular phylogenetic analyses revealed the presence of three lineages corresponding to three geographically disjunctive populations of *C. fulguratus* in Thailand. Likelihood tests of topologies significantly supported the non-monophyly of the *C. fulguratus*–complex and Bayesian species delimitation analysis significantly supported the potential representation as distinct species of these three lineages. Discriminant function analysis based on geometric-morphometrics of shell shape allowed for significant distinction of these three candidate species, although they revealed a considerable degree of overlap of shell shape reflecting their crypsis morphologically. The diagnostic characters are provided by color pattern, pattern of protoconch and pattern of jaw. In conclusion, the results support that the *C. fulguratus* s.l., as currently recognized, consists of three distinct species in Thailand: *C. fulguratus* s.s., *C. rangunensis* and *C. abditus* sp.nov., which are described herein.

## Introduction

*Cyclophorus* Monfort, 1810 is a genus of operculate land snail distributed throughout the humid or seasonally humid tropical and warm temperate habitats of South Asia and South East Asia, as well as the southern areas of China, Korea, and Japan [1]–[9]. It is one of the most diverse genera of land snails, comprising more than 100 nominal species, and is among the vaguest in terms of its taxonomy [2], [3]. *Cyclophorus* have been utilized for food in many parts of Thailand, Laos, and Vietnam [10]–[12], but in recent years, the number of *Cyclophorus* snails appears to have noticeably decreased [13]. The traditional classification of *Cyclophorus* is based solely on shell morphological characters such as shape and color pattern [1]–[3], [6]. However, shell characteristics can be extremely variable, especially in widely distributed species, due to convergence [14]–[18] or random genetic drift and geographic isolation [19], [20]. Some studies have investigated the anatomy of *Cyclophorus* [21], [22] but it is quite clear that *Cyclophorus* shows a high degree of anatomical similarity rendering anatomical characters unreliable for use in species discrimination [23], [24].

*Cyclophorus fulguratus* (Pfeiffer, 1854) is characterized by a turbinated shell that is transversely freckled with zigzag chestnut streaks, conspicuously banded below the periphery, and with a circular aperture with a white or pale orange lip [25]. It has a widespread distribution throughout Southeast Asia, including Cambodia, Laos, Myanmar, Thailand, and Vietnam [2], [3]. In Thailand, it was recorded in the western, central, eastern, and northeastern parts of the country. More recently, studies on karyotype [23], allozyme analysis [26], and molecular phylogeny [27] have revealed extensive variation within Thai *C. fulguratus* populations. These genetic variations, in combination with the high degree of intra-specific shell variation in *C. fulguratus*, would suggest that its classification as a single species warrants reconsideration [23], [26]. The current taxonomic focus on key external shell characteristics, which have been shown in several cases to be strongly influenced by environmental factors, may confound the identification of useful diagnostic characters for the recognition of biological species of *Cyclophorus* [16], [26], [27]. Species limits within *Cyclophorus* are extremely difficult to establish, with geographically disjunctive populations of *C. fulguratus* perhaps representative of a complex of cryptic species [27].

The taxonomic status of *C. fulguratus* remains confused and there is a clear need to reassess its taxonomy through the use of more effective tools. This paper aims to determine the validity of species boundaries in *C. fulguratus* by combining molecular phylogeny and morphological approaches. The recognition of cryptic species allows these taxa to be included in biodiversity assessments and incorporated in conservation strategies.

## Material and Methods

### Taxon sampling and morphological study

One hundred and fifty-five *C. fulguratus*, including live and shell-only specimens, were collected from 34 localities throughout its distribution range in Thailand. The identification of specimens was primarily based on publications of Pfeiffer (1854) [25], and Kobelt (1902, 1908) [2], [3]. All specimens were then subsequently compared with the relevant type specimens from the Natural History Museum, London (NHMUK) [28]. As the adult specimens exhibit an expanded and reflexed apertural lip they are easy to distinguish. Foot tissue were fixed and preserved in 95% ethanol for molecular study. The remaining parts were preserved in 70% ethanol for anatomical study. All of the voucher specimens were kept at the Chulalongkorn University Museum of Zoology (CUMZ), Bangkok, Thailand (voucher numbers are given in Table 1).

**Table 1:** List of collecting site of *Cyclophorus fulguratus*, *C. rangunensis* and *C. abditus* sp. nov., voucher numbers (CUMZ) and the accession no. of 18S rRNA, 28S rRNA, 16S rRNA and COI genes.

Species/Locality	GPS coordinates	CUMZ nos.	Number of specimens		GenBank Accession No.			
			DNA	Morphometric	18S rRNA	28S rRNA	16S rRNA	COI
*Cyclophorus fulguratus* s.s.								
1. Khao Chakan, Srakaeo	102° 05′ 01.94″ E, 13° 39′ 36.96″ N	1327	3	13	KJ407135-7	KJ407220-3	KJ407181-3	KJ407259-61
2. Khao Maka Cave, Srakaeo	102° 42′ 39.2″ E,12° 6′ 8.1″ N	1688	3	5	KJ407138-40	KJ407223-5	KJ407184-7	KJ407262-4
3. Sapanhin waterfall, Trat	1 02° 42′ 39.2″ E, 12° 6′ 8.1″ N	1614	3	4	KJ407132-4	KJ407217-9	KJ407178-80	KJ407256-8
4. Plieu National Park, Chanthaburi	102° 10′ 11″ E, 12° 31′ 05″ N	822, 863, 1180	-	12	-	-	-	-
5. Makok Waterfall, Chanthaburi	102° 15′ 14″ E, 12° 35′ 08″ N	1135	-	8	-	-	-	-
6. Khao Soi Dao Waterfall, Chanthaburi	102° 11′ 36″ E, 13° 06′ 15″ N	1076	-	6	-	-	-	-
7. Khao Sukim Temple, Chanthaburi	102° 01′ 56″ E, 12° 45′ 58″ N	1224	-	2	-	-	-	-
*Cyclophorus rangunensis*								
8. Srisatchanarai, Sukhothai	99° 46′ 57.09″ E, 17° 25′ 59.70″ N	1430	2	5	KJ407095-6	KJ407188-9	KJ407149-50	KJ407227-8
9. Ramkamhaeng National Park, Sukhothai	99° 41′ 44.20″ E, 16° 52′ 34.88″ N	1189	2	5	KJ407097-8	KJ407190-1	KJ407151-2	KJ407229-30
10. Thepsatit Temple, Nakhonsawan	99° 54′ 51.07″ E, 15° 57′ 55.34″ N	809	2	5	KJ407099-100	KJ407192-3	KJ407153-4	KJ407231-2
11. Thepsataporn Temple, Thap Than, Uthaithani	99° 43′ 51.89″ E, 15° 27′ 20.10″ N	1232	2	5	KJ407101-2	KJ407194-5	KJ407155-6	KJ407233-4
12. Klong Lan waterfall, Kamphaeng Phet	99° 16′ 37.5″ E, 16° 7′ 50.4″ N	1602	2	4	KJ407105-6	KJ407197, KF319146*	KJ407158, JX474708*	KJ407236, JX474582*
13. Thepmuangthong Temple, Lan Sak, Uthaithani	99° 35′ 37″ E, 15° 24′ 59″ N	1781	2	4	KJ407103-4	KJ407196, KF319143*	KJ407157, JX474705*	KJ407235, JX474579*
14. Khao Bin Cave, Chom Bueng, Ratchaburi	99° 40′ 00″ E, 13° 35′ 36″ N	1660	2	2	KJ407107-8	KJ407198, KF319145*	KJ407159, JX474707*	KJ407237, JX474581*
15. Doi Haumod Mountain, Umphang, Tak	98° 51′ 22.1″ E, 15° 57′ 36.5″ N	1747	2	5	KJ407109-10	KJ407199, KF319144*	KJ407160, JX474706*	KJ407238, JX474580*
16. Khao Noh, Nakhon Sawan	99° 52′ 05″ E, 15° 56′ 01″ N	1064	-	3	-	-	-	-
17. Pha Subin, Nakhon Sawan	100° 22′ 8″ E, 15° 16′ 25″ N	1164	-	4	-	-	-	-
18. Khao Nang Rum, Uthaithani	99° 28′ 21″ E, 15° 12′ 45″ N	1062, 1063	-	4	-	-	-	-
19. Bhumibol Dam, Tak	99° 01′ 25″ E, 17° 15′ 27″ N	1176	-	5	-	-	-	-
20. Tam Sue Temple, Suphanburi	99° 51′ 09″ E, 14° 21′ 07″ N	1401	-	2	-	-	-	-
*Cyclophorus abditus* sp. nov.								
21. Nawa, Nakhon Phanom	104° 04′ 12.46″ E, 17° 34′ 00.76″ N	1399	2	5	KJ407113-4	KJ407201-2	KJ407162-3	KJ407240-1
22. Lumpahung, Sakon Nakhon	103° 49′ 36.46″ E, 17° 27′ 29.88″ N	1750	2	5	KJ407115-6	KJ407203-4	KJ407164-5	KJ407242-3
23. Nanghong cave, Sakon Nakhon	103° 40′ 9.47″ E, 17° 19′ 17.84″ N	1690	2	5	KJ407117-8	KJ407205-6	KJ407166-7	KJ407244-5
24. Waritchaphum, Sakon Nakhon	103° 37′ 55.81″ E, 17° 13′ 37.64″ N	1838	2	5	KJ407119-20	KJ407207-8	KJ407168-9	KJ407246-7
25. Nam-un Dam, Sakon Nakhon	103° 45′ 26.66″ E, 17° 18′ 14.21″ N	1839	2	4	KJ407121-2	KJ407209-10	KJ407170-1	KJ407248-9
26. Namphung Dam, Sakon Nakhon	103° 55′ 35.71″ E, 16° 58′ 12.81″ N	1145	2	3	KJ407123-4	KJ407211-2	KJ407172-3	KJ407250-1
27. Namlad, Sakon Nakhon	103° 56′ 1.68″ E, 16° 50′ 58.27″ N	1840	2	3	KJ407125-6	KJ407213-4	KJ407174-5	KJ407252-3
28. Phu Kum Khao, Kalasin**	103° 31′ 25.72″ E, 16° 41′ 44.67″ N	1828	2	3	KJ407127-8	KJ407215-6	KJ407176-7	KJ407254-5
29. Tum Numthip Temple, Roiet	104° 19′ 05″ E, 16° 19′ 15″ N	1826	1	3	KJ407130	KF319183*	JX474701*	JX474619*
30. Phu Phan cave, Sakon Nakhon	103° 58′ 11.8″E, 17° 05′ 42″ N	1609	1	2	KJ407131	KF319182*	JX474700*	JX474618*
31. Nakhon Phanom to Dong Luang, Mukdahan	104° 30′ 45″ E, 16° 56′ 38″ N	1751	1	3	KJ407129	KF319181*	JX474699*	JX474617*
32. Kang Lumduan waterfall, Ubon Ratchathani	105° 06′ 29.1″ E, 14° 26′ 11″ N	1827	2	2	KJ407111-2	KJ407200, KF319186*	KJ407161, JX474704*	KJ407239, JX474622*
33. Phu Pha Man, Konkean	101° 54′ 03″ E, 16° 40′ 25″ N	1171	-	3	-	-	-	-
34. Phu Khiao, Chaiyaphum	102° 07′ 43″ E, 16° 19′ 29″ N	1254	-	3	-	-	-	-
35. Ban Dong Kum Pho, Sakon Nakhon	103° 43′ 26″ E, 17° 17′ 11″ N	1005	-	3	-	-	-	-
*Cyclophorus affinis*	98° 55′ 45.3″ E, 19° 23′ 40.8″ N	1777	1	-	KJ407081	KF319151*	JX474678*	JX474587*
*Cyclophorus amoenus*	99° 52′ 02″ E, 15° 56′ 00″ N	1654	1	-	KJ407086	KF319160*	JX474661*	JX474596*
*Cyclophorus aurantiacus*	98° 17′ 43″ E, 8° 38′ 49″ N	1769	1	-	KJ407088	KF319206*	JX474723*	JX474642*
*Cyclophorus bensoni*	101° 11′ 54.1″ E, 19° 11′ 25″ N	1833	1	-	KJ407082	KF319138*	JX474670*	JX474574*
*Cyclophorus cantori*	98° 59′ 5.0″ E, 9° 1′ 42.2″ N	1697	1	-	KJ407089	KF319193*	JX474718*	JX474629*
*Cyclophorus consociatus*	103° 12′ 31″ E, 16° 49′ 29″ N	1606, 1750	2	-	KJ407084-5	KF319184-5*	JX474702-3*	JX474620-1*
*Cyclophorus courbeti*	102° 13′ 07″ E, 13° 59′ 36″ N	1613	1	-	KJ407080	KF319175*	JX474693*	JX474611*
*Cyclophorus cryptomphalus*	99° 56′ 0.3″ E,15° 44′ 46.4″ N	1741	1	-	KJ407078	KF319158*	JX474665*	JX474594*
*Cyclophorus diplochilus*	98° 59′ 13″ E, 9° 01′ 47″ N	1712	1	-	KJ407090	KF319191*	JX474714*	JX474627*
*Cyclophorus expansus*	99° 21′ 28″ E, 10° 59′ 27″ N	1723	1	-	KJ407094	KF319195*	JX474720*	JX474631*
*Cyclophorus haughtoni*	101° 43′ 42″ E, 12° 55′ 25″ N	1624	1	-	KJ407079	KF319179*	JX474697*	JX474615*
*Cyclophorus labiosus*	101° 21′ 36.2″ E, 14° 31′ 33.3″ N	1791	1	-	KJ407087	KF319174*	JX474692*	JX474610*
*Cyclophorus malayanus*	99° 18′ 18.9″ E, 14° 39′ 29.5″ N	1683	1	-	KJ407092	KF319134*	JX474654*	JX474570*
*Cyclophorus pfeifferi*	98° 35′ 28″ E, 16° 50′ 14″ N	1779	1	-	KJ407093	KF319155*	JX474683*	JX474591*
*Cyclophorus saturnus*	100° 25′ 18.3″ E, 17° 35′ 39 ″ N	1775	1	-	KJ407083	KF319130*	JX474674*	JX474566*
*Cyclophorus speciosus*	102° 10′ 26.3 ″ E, 12° 34′ 58″ N	1619,	2	-	KJ407145-6	KF319139-40*	JX474655-6*	JX474575-6*
*Cyclophorus subfloridus*	101° 08′ 32″ E, 16° 40′ 40″ N	1768, 1770	2	-	KJ407143-4	KF319164-5*	JX474666-7*	JX474600-1*
*Cyclophorus volvulus*	99° 49′ 18″ E, 11° 51′ 39.2″ N	1806	2	-	KJ407141-2	KF319147-8*	JX474709-10*	JX474583-4*
*Cyclophorus zebrinus*	98° 36′ 7.6″ E, 15° 1′ 39″ N	1743	1	-	KJ407091	KF319196*	JX474721*	JX474632*
*Leptopoma vitrium*	102° 15′ 14″ E, 12° 35′ 08″ N	1839	2	-	KJ407147-8	KJ407226, KF319214*	KJ407187, JX474741*	KJ407265, JX474650*

*sequences from Nanatarat et al. (2014) [27].

**type locality of the *Cyclophorus abditus* sp. nov.

For details of shell surface, protoconch, jaw and radula morphology, the samples were coated with gold metal and examined in a scanning electron microscopy (SEM, JSM-5410 LV) at the Scientific and Technological Research Equipment Centre (STREC), Chulalongkorn University.

### Molecular phylogeny

**Figure 1:**
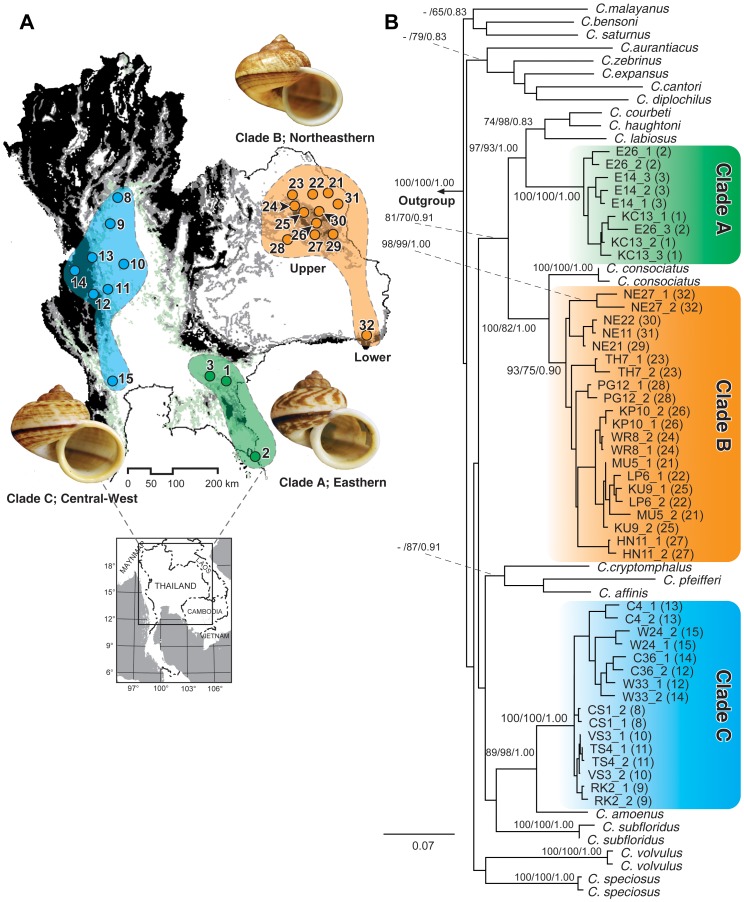
**(A)** Location of *Cyclophorus fulguratus* species complex sampling sites in Thailand. The numbered sample sites are detailed in Table 1. **(B)** Maximum-likelihood phylogenetic tree of the *Cyclophorus fulguratus* species complex and related species constructed using 2146 nucleotide sites of the concatenated 18S, 28S, 16S and COI genes. Bootstrap support values (when>65%) and posterior probabilities for individual nodes are shown on the tree (based on NJ/ML/BI methods).

For molecular analysis, the 46 live specimens of *C. fulguratus* from 23 localities, including the voucher specimens from previous studies of Kongim et al. (2006) [23], Prasankok et al. (2009) [26], and Nantarat et al. (2014) [27], were used (Fig. 1,Table 1). Nineteen congeners of the genus *Cyclophorus* found in Thailand were also included. *Leptopoma vitrium* was used as the outgroup.

Genomic DNA was extracted from foot tissues by using a DNAeasy Tissue Kit (QIAGEN Inc.). Sequence data was obtained for the nuclear 18S rRNA (18S; approximately 440 bp, positions 1391-1845 of the complete 18S rRNA gene of ‘*Biomphalaria glabrata*’ GB accession number U65223) and 28S rRNA (28S; approximately 590 bp, position 213-853 of the partial 28S rRNA gene of ‘*Architectonica perspectiva*’ GB accession number FJ917231.1) as well as the mitochondrial 16S rRNA (16S; approximately 518 bp, positions 672-1198 of the complete 16S rRNA gene of ‘*Oncomelania hupensis*’ GB accession number NC_013073.1) and cytochrome c oxidase I (COI; 660 bp, positions 37-696 of the complete COI gene of ‘*Oncomelania hupensis*’ GB accession number NC_013073.1) gene fragments.

For each gene fragment, amplification was attempted with a single pair of primers as in Table 2. PCR reactions were undertaken in a 50 µl final volume using 25 µl of 2xIllustra hot start master mix (GE Healthcare), 10 µM of each primer and about 10 ng of DNA template. The 18S, 28S, and 16S PCR reaction cycles were as follows: 2 min at 94°C, subsequently 36 cycles of 30 sec at 94°C, 30 sec at 50°C, and 90 sec at 72°C, and then a final extension step of 5 min at 72°C. For COI, PCR reaction cycles were 2 min at 94°C, subsequently 36 cycles of 30 sec at 94°C, 2 min at 42°C, and 2 min at 72°C, and then a final extension step of 5 min at 72°C. The PCR products were purified with a QIAquick PCR purification Kit (QIAGEN Inc.) and sequences were sent for cycle-sequencing at Macrogen, Inc.

**Table 2:** List of primers used in this study.

Gene	Primer name	Primer sequence (5′–3′)	References
18S	A1	TTACTCGATGCGACGGCGC	This study
	9R	GATCCTTCCGCAGGTTCACCTAC	[61]
28S	28SF4	AGTACCGTGAGGGAAAGTTG	[62]
	28SR5	ACGGGACGGGCCGGTGGTGC	[62]
16S	16sar	CGCCTGTTTATCAAAAACAT	[63]
	16sbr	CCGGTCTGAACTCAGATCACGT	[63]
COI	LCO1490	GGTCAACAAATCATAAAGATATTGG	[64]
	HCO2198	TAAACTTCAGGGTGACCAAAAAATCA	[64]

Sequences were initially aligned in MUSCLE version 3.6 [29], with manual adjustments made in MEGA 5.0 [30]. Ambiguously aligned nucleotide positions were identified manually and excluded from sequence analyses [31]. All gaps were likewise excluded. All base frequencies, pairwise (uncorrected-p) sequence distances for all gene fragments and molecular character statistics were calculated using MEGA 5.0 [30]. Heterogeneity in base composition between sequences was tested by examining the variation in base pair composition among sequences for all datasets using a χ2 analysis, as implemented in PAUP* v4.0b10 [32].

Neighbor-joining (NJ), maximum likelihood (ML), and Bayesian inference (BI) were performed using the following datasets: 18S, 28S, 16S, COI, and concatenated 18S, 28S, 16S and COI. The concatenated dataset (Material S1) was assessed for conflict between the character partitions using the partition homogeneity test in PAUP 4.0b10, using 100 replicates [32], [33]. The data were partitioned by gene fragment with the evolutionary substitution model specified for each partition separately. jModeltest 0.1.1 [34] was used to calculate the best evolutionary substitution model under Akaike Information Criterion; AIC [35].

NJ analysis was undertaken using PAUP* v4.0b10 [32] based on appropriate models (18S, HKY+I+G; 28S, HKY; 16S, GTR; COI, GTR+I+G; concatenated dataset, GTR+G). Bootstrap resampling [36] with 1000 replicates was undertaken to assign support to branches in the NJ tree. For ML and BI analyses, single gene analyses were undertaken using the following models: 18S, HKY+I+G; 28S, HKY; 16S, GTR; COI, GTR+I+G. For the concatenated dataset a partitioned analysis was performed. ML analyses was performed using RAxML v7.2.6 [37], with randomized stepwise addition parsimony trees (number seed  = 5), and with 1000 bootstrap replicates [36] via the rapid bootstrap procedure of Stamatakis et al. (2008) [38]. BI analysis was performed using MrBayes version 3.1.2 [39], where the tree space was explored using four chains for each run of a Markov chain Monte Carlo algorithm (MCMC) and the optimum substitution model suggested by jModeltest [34]. The BI analysis was run for 10,000,000 generations (heating parameter  = 0.03), sampled every 100 generations, and the last 10,000 of trees were used for calculation of posterior probabilities on the Bayesian inference tree (burnin = 90,001samples). Convergence was monitored by proving the average standard deviation of the split frequencies (between 2 runs) were below 0.01 [39].

Alternative phylogenetic hypotheses of the polyphyly of *C. fulguratus* were assessed using a Shimodaira and Hasegawa test (SH-test) [40] and an Approximately Unbiased test (AU-test) [41]. The SH and AU tests were implemented in the program CONSEL version 0.1i [42] with 10,000 bootstrap replicates. The SH and AU tests were used to compare likelihood scores of constraint trees in which *C. fulguratus*–complex monophyly was enforced against the tree inferred by the ML method. The constraint topologies and site-likelihood scores were calculated in RAxML with the –g constraint option using the best scoring tree for the topology tests. The trees were estimated from 300 ML searches starting from random tree topologies. Constraints tested were: (i) monophyly of Clade A, B and C (ii) monophyly of Clade A and B, (iii) monophyly of clade A and C, and (iv) monophyly of Clade B and C.

**Figure 2:**
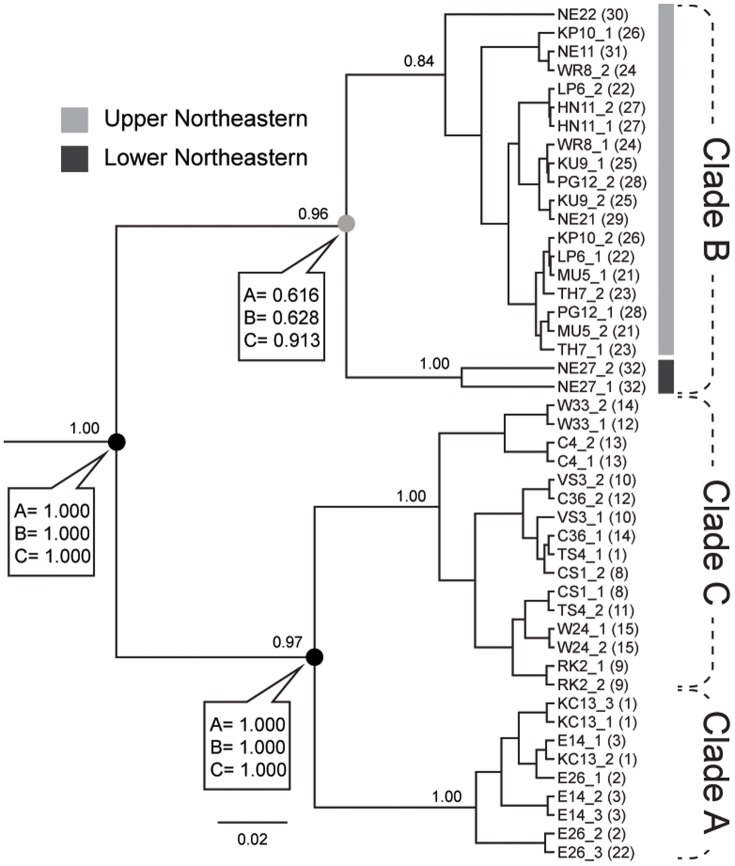
*BEAST species tree inference output based on all loci for the *C. fulguratus* – complex. Black nodes show 95% speciation probability support values in Bayesian species delimitation; Gray nodes are not supported in over 95% by Bayesian species delimitation. Posterior probability for supported branches generated by *BEAST are shown above the branches and speciation probabilities of a Bayesian species delimitation are provided in box for each node under each combination of priors: A, relatively large ancestral population size and shallow divergences; B, relatively large ancestral population size and deep divergences; and C, relatively small ancestral population size and shallow divergences. The 95% of speciation probability value is considered as strong support for a speciation event and is shown for each node.

In addition, the validity of the three clades (Fig. 1; see results) was also determined by using a Bayesian species-delimitation approach (BSD), as implemented in the program Bayesian Phylogenetics and Phylogeography (BP&P) version 2.2 [43]. A total of 46 samples of *C. fulguratus* s.l. were included. BSD is based on the reversible-jump Markov Chain Monte Carlo (rjMCMC) algorithm in combination with a user-specified guide tree. The tree topology from a species tree based on concatenated sequences of 18S, 28S, 16S and COI genes generated from *BEAST [44], part of the BEAST v1.6.1 package [45], was used as a guide tree. The species trees (Fig. 2) were calculated for the *C. fulguratus*–complex using the same models as used in the ML and BI methods. Analyses were run with unlinked substitution model and clock model parameters (using same settings as for gene tree analyses). The Yule process was performed as the species tree prior. The two independent runs with a MCMC chain length of 1×10^7^ each were conducted, sampling every 1000 generations. Convergence and effective sample sizes (ESS) were assessed with Tracer v1.5 [46] before building a consensus tree with 10% of burn-in. We used algorithm 0 with the fine-tuning parameter e = 5 to adjust the rjMCMC and ran 1×10^6^ generations with a sampling every 100 and a burn-in of 10%. The 95% of speciation probability value is considered as strong support for a speciation event [47]. Three different combinations of ancestral population size (θ) and root divergence time (τ) as suggested by Ahmadzadeh et al. 2013 [48] were evaluated: (A) relatively large ancestral population size and shallow divergences, θ = G(θ 2,10), τ = G(θ 2,2000); (B) relatively large ancestral population size and deep divergences, θ = G(θ 2,10), τ = G(θ 2,10); and (C) relatively small ancestral population size and shallow divergences θ = G(θ 2,2000), τ = G(θ 2,2000). For ensuring stability, we ran each analysis three times with proper mixing and convergence across runs as discussed above.

### Shell geometric morphometric analysis

**Figure 3:**
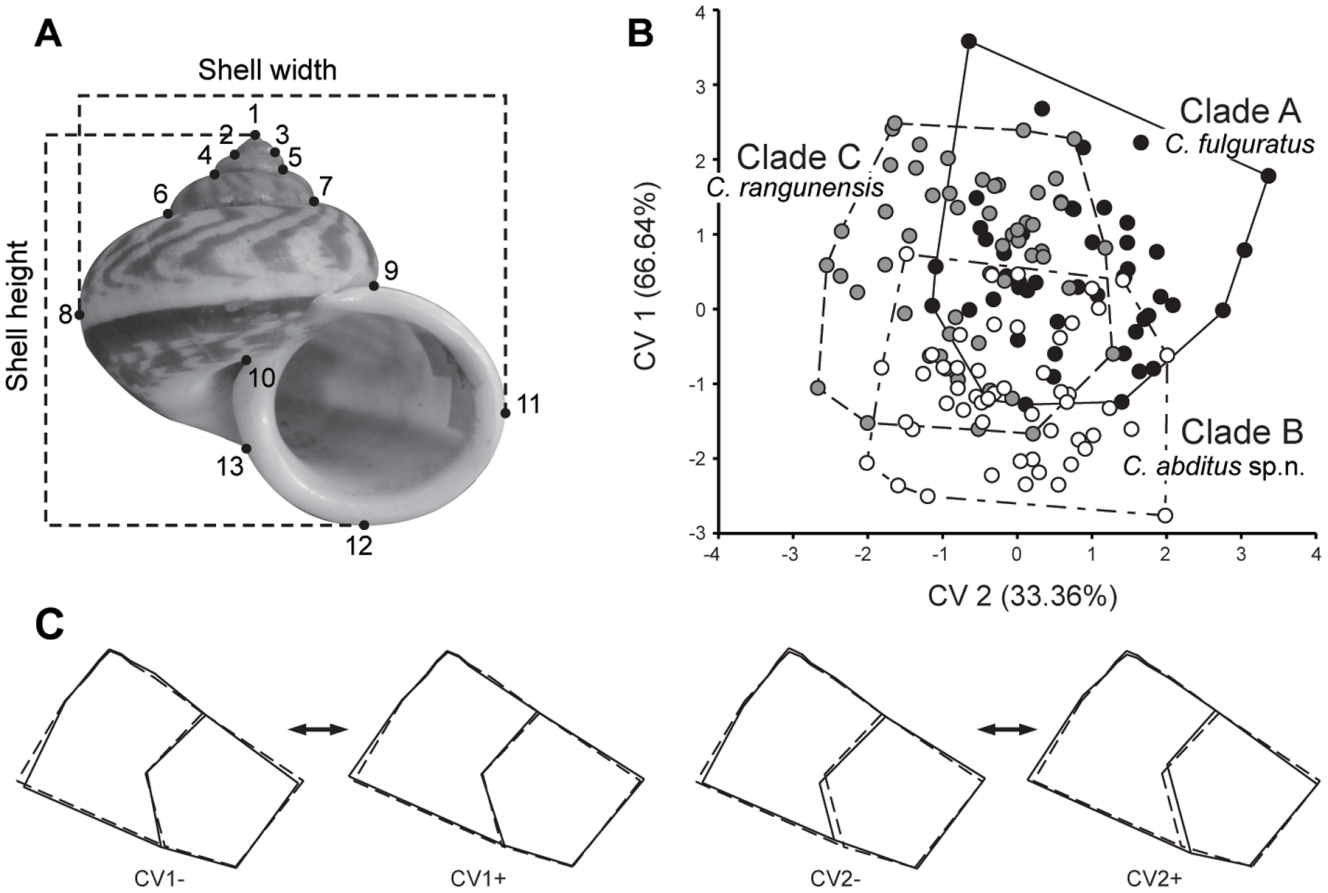
Geometric-morphometric study of shell shape variation in *Cyclophorus fulguratus* s.l. **(A)** Shell photo showing measurements and landmarks (black circles) used in this study. **(B)** Plots of individual scores for the two canonical variates (CVs) derived from canonical variate analysis (CVA). Black circles represent individuals for Clade A, white circles for Clade B and grey circles for Clade C. **(C)** Wireframes showing the shape deformations (solid line) from the consensus configuration (dotted line) to each extreme negative and positive CVs. Shape changes along CV1 are shown on the left and CV2 on the right.

For geometric morphological study, 155 shells, including the shell-only collections as well as the individuals for which we gathered DNA sequence data, were examined. Shell photos were taken with a tripod-mounted Nikon D90 digital camera using consistent capture conditions for all specimens. The photos were randomly ordered in tpsUtil v.1.49 [49], before bi-dimensional coordination of thirteen landmarks shown as Fig. 3A were digitized by the same person in the program tpsDig2 v.2.16 [50].

Geometric morphometrics analyses were carried out using the software package MORPHOJ v1.05c [51]. Landmark data were first superimposed by Procrustes transformation into a common coordinate system to eliminate other variation (i.e. isometric size variation and orientation) except shape from the data [52]. The allometric components or the effect of allometric shape variation were removed. Shell shapes as the independent variation were plotted against centroid size as the independent variation. Multivariate regression revealed a statistically significant correlation between the centroid size and shape variable (p<0.001; 10,000 replicates of permutation test), and accounted for 5.58% of total variation. Therefore, to remove this allometric component, a new data set that contained the residual was used in subsequent analyses.

Shell shape variation was then examined using canonical variance analysis (CVA) with candidate species recognized by molecular analyses as *a prior* group. Mahalanobis distances and Procrustes distances between pairwise groups were assessed for significant differences by the permutation test (10 000 iterations). The percentage of correct classification of a paired species was assessed by leave-one-out cross-validation as the implement of discriminant function analysis.

### Ethical considerations

All animal work was conducted according to relevant national and international guidelines. No specific permissions are required to work with invertebrates in Thailand. Similarly, for the majority of collection sites, no specific permissions were required for the collection of snails, because they were not collected from protected areas of land. Where specimens were collected from national parks, collections were undertaken in cooperation with the Plant Genetic Conservation Project initiated by Her Royal Highness Princess Maha Chakri Sirindhorn and the Ministry of Natural Resources and Environment (MNRE) (permit number for 2553-2555). The land snail, *C. fulguratus*, is not an endangered or protected species.

### Nomenclatural acts

The electronic edition of this article conforms to the requirements of the amended International Code of Zoological Nomenclature, and hence the new names contained herein are available under that Code from the electronic edition of this article. This published work and the nomenclatural acts it contains have been registered in ZooBank, the online registration system for the ICZN. The ZooBank LSIDs (Life Science Identifiers) can be resolved and the associated information viewed through any standard web browser by appending the LSID to the prefix “http://zoobank.org/”. The LSID for this publication is: urn:lsid:zoobank.org:pub: F8E64879-4502-48DB-9B4C-10EE87B58C87.

## Results

### DNA sequence variation

A total of 46 samples from 23 populations of *C. fulguratus* throughout Thailand were sequenced. The aligned matrices include two nuclear gene fragments, 18S (431 bp) and 28S (585 bp), and two mitochondrial gene fragments, 16S (470 bp) and COI (660 bp). Variable/parsimony-informative characters are: 150/100 (18S); 52/31 (28S); 150/117 (16S); and 273/239 (COI).

The concatenated dataset included a total of 2146 aligned characters. The dataset had 46.2% GC content [range 41.09% GC to 46.61% GC, χ2 test = 27.148126 d.f. = 153, P = 1.000], with 754 parsimony informative and 791 variable sites. The results of a partition homogeneity test by PAUP 4.0b10, using 100 replicates [32] showed no significant differences in the sequences were found between markers (P = 0.096). The uncorrected p-distance between the taxa ranged from 0.121 to 0.144 (inter/intraspecific p-distances = 0.067 and 0.007, respectively).

### Molecular phylogeny

The phylogenetic tree based on the concatenated data set of all genes (Fig. 1B) showing the evolutionary relationships among populations of the *C. fulguratus*–complex and their position among congeners resulted in topologies with all branches supported with>65% of NJ/ML bootstraps and>0.83 of BI posterior probabilities. The inferred topologies strongly support the polyphyly of the *C. fulguratus*–complex, with this finding consistent for all genes and across all tree methods. The *C. fulguratus*–complex is separated into three clearly defined clades that correspond well to biogeographic regions (Fig. 1). The three clades consisted of Clade A, the Eastern population with 100% NJ and 100% ML bootstrap support and a Bayesian posterior probability of P = 1.00; Clade B, the Northeastern population with 93% NJ and 75% ML bootstrap support and P = 0.90 (BI); and Clade C, the Central-West population with 100% NJ and 100% ML bootstrap support and P = 1.00 (BI). Clade A was placed closely to *C. coubeti*, *C. haughtoni*, and *C. labiosus* in the phylogenetic tree, while Clade B was clustered with *C. consociatus*, and Clade C appeared as a sister group with *C. amoenus* and *C. subfloridus* (Fig. 1B).

Both SH and AU tests significantly reject the monophyly of the *C. fulguratus*–complex in all of the analyzed datasets (18S, 28S, 16S, COI, concatenated 18S and 28S, concatenated 16S and COI, and concatenated 18S, 28S, 16S and COI) at p<0.01 [monophyletic *C. fulguratus* (all clades) against inferred tree]. Constraint trees in which the monophyly of paired clades were tested were also significantly rejected in all analyzed datasets at p<0.01 [(i) monophyly of Clade A and B against inferred tree, (ii) monophyly of clade A and C against inferred tree, and (iii) monophyly of Clade B and C against inferred tree].

Bayesian species delimitation strongly supports three species within the *C. fulguratus* –complex (Clade A, B, C; Fig.2). These were consistently recovered in the posterior distribution by every species delimitation model (speciation probability  = 1) under every prior combination (all effective sample sizes, parameters above 200). Clade B was further divided into two subclades, which are distributed in the northern (Upper) and southern (Lower) parts of the northeastern range. However, these were not significantly delimited as distinct species in Bayesian species delimitation analysis (prior A, speciation probability  = 0.616; prior B, 0.628; and prior C, 0.913).

The distance for the 18S, 28S, 16S and COI gene fragments between all *Cyclophorus* species included in this analysis (excluding outgroup; Table 3) and between the three *C. fulguratus* clades (Tables 4–5) also supported the presence of three clades of *C. fulguratus*.

**Table 3:** Ranges of genetic divergence for the 18S, 28S, 16S and COI gene fragments between all *Cyclophorus* species included in this analysis (excluding outgroup).

Distance	18S	28S	16S	COI
p-distance	0.0–8.1% (average 3.6%)	0.0–4.1% (average 1.2%)	0.0–10.6% (average 6.8%)	0.0–17.6% (average 10.9%)
corrected distance*	0.0–10.9% (average 4.0%)	0.0–4.4% (average 1.3%)	0.0–11.8% (average 7.4%)	0.0–21.3% (average 12.4%)

*18S, HKY+I+G; 28S, HKY; 16S, GTR; COI, GTR+I+G.

**Table 4:** Ranges of genetic divergence of three *Cyclophorus fulguratus* clades and related species based on 18S rRNA gene (above diagonal) and 28S rRNA (below diagonal).

	Clade A	Clade B	Clade C
Clade A: *C. fulguratus*	-	0.085 (0.087)	0.067 (0.069)
Clade B: *C. abditus* sp.nov.	0.014 (0.015)	-	0.039 (0.041)
Clade C: *C. rangunensis*	0.010 (0.012)	0.015 (0.016)	-

*corrected distance in bracket based on model as follows: 18S, HKY+I+G; 28S, HKY.

**Table 5:** Ranges of genetic divergence of three *Cyclophorus fulguratus* clades and related species based on 16S rRNA gene (above diagonal) and COI gene (below diagonal).

	Clade A	Clade B	Clade C
Clade A: *C. fulguratus*	-	0.071 (0.075)	0.093 (0.098)
Clade B: *C. abditus* sp.nov.	0.097 (0.099)	-	0.094 (0.098)
Clade C: *C. rangunensis*	0.114 (0.125)	0.117 (0.137)	-

*corrected distance in bracket based on model as follows: 16S, GTR; COI, GTR+I+G.

### Geometric morphometric analysis

Canonical variance analysis (CVA) with the three clades of the *C. fulguratus*–complex given as *a prior* group provided a graphic display of the shape differences between them by the relation between two CV variables obtained (Fig. 3B). The first canonical axis (CV1) explained 66.64% (Eigenvalues  = 0.6858) of the shape variability, while the second canonical axis (CV2) accounted for 33.36% (Eigenvalues  = 0.3432). Overall, individuals from each clade were clustered with a considerable overlap with other clades. When testing for differences among clades, however, the results showed significant differences among them based on permutation tests for Mahalanobis distances and Procrustes distances (*P*<0.0001; to 0.0013: Table 6). The percentage of correct classification of specimens according to cross-validation of discriminant function analysis was 66% for Clade A; 79% for Clade B; and 65% for Clade C. The overall rate of reliability in the identification of specimens was 70%.

**Table 6:** Mahalanobis distances and Procrustes distances of three *Cyclophorus fulguratus* clades derived from canonical variate analysis (CVA) of the shell shape with *p-values* (shown in parenthesis) calculated by 10,000 random permutations per test to determine statistical significance of differences between pairs of clades.

	Clade A	Clade B	Clade C
Clade A: *C. fulguratus*	-	1.8367 (<0.0001)	1.4306 (<0.0001)
Clade B: *C. abditus* sp.nov.	0.0216 (<0.0001)	-	1.9097 (<0.0001)
Clade C: *C. rangunensis*	0.0187 (0.0003)	0.0165 (0.0013)	-

Above diagonal is Mahalanobis distances; below diagonal is Procrustes distances.

Wireframe modification in Figure 3C described the shape change from the consensus configuration along the two CVs. For CV1, individuals located in the positive portion of the axis had a wider shell when compared to individuals located in the negative portion. This shape change was principally represented by a shift of landmark 8 and 11. The CV2 showed differences in relation to the aperture of the snails. Individuals placed in the positive portion of the axis had a narrower aperture width when compared to the negative portion. Shape deformations along CV2 were markedly described by landmark 10 and 13 moving from the center of the aperture with respect to other landmarks as the score decreased.

### Morphological data

**Figure 4:**
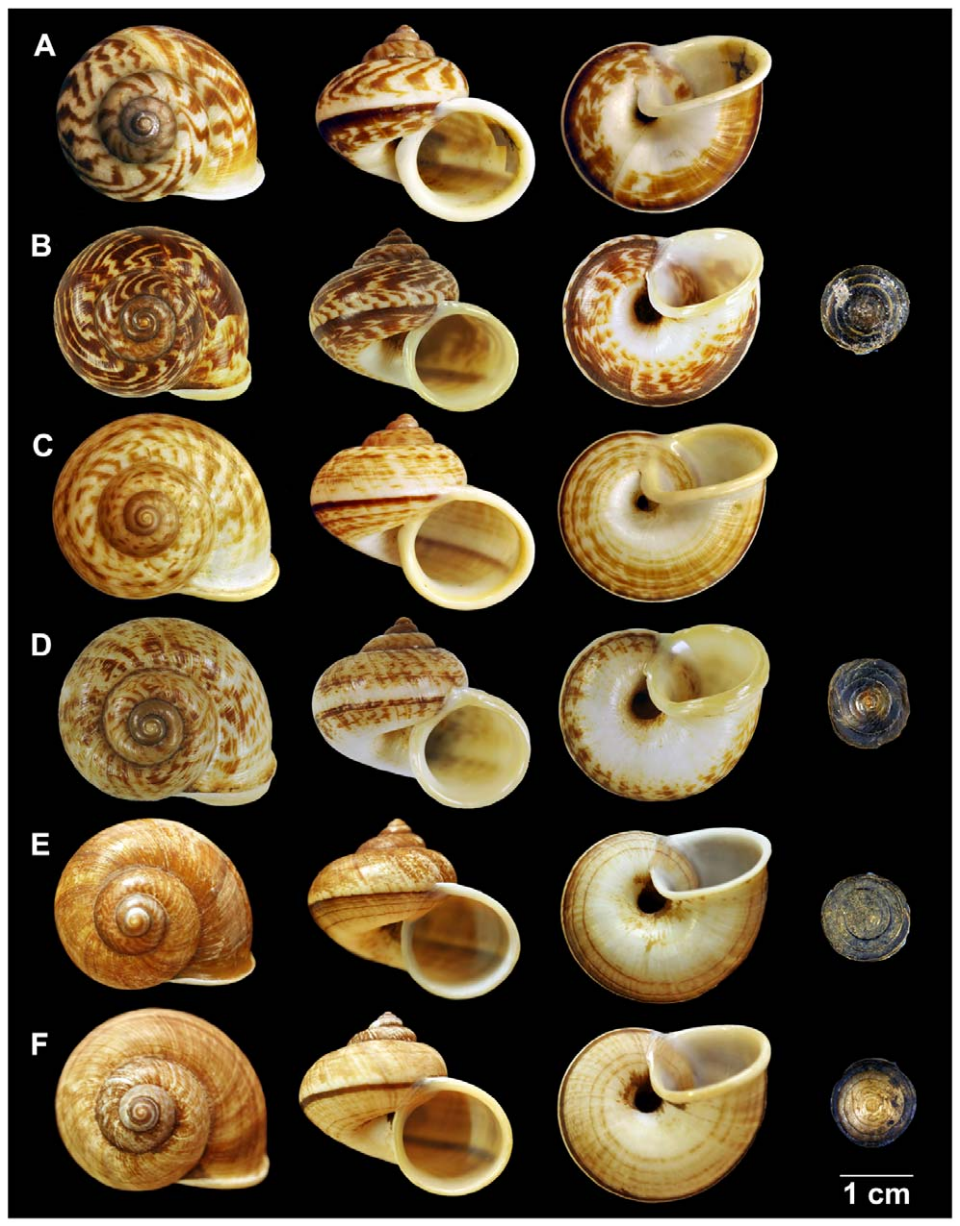
Shell of *Cyclophorus* species. **(A, B)** *Cyclophorus fulguratus* s.s. **(A)** Lectotype NHMUK 20130117/1, and **(B)** specimen CUMZ 1327 from Khao Chakan, Sra Kaeo (Clade A; Fig. 1B). **(C, D)** *Cyclophorus rangunensis* **(C)** Lectotype NHMUK 20130091/1, and **(D)** specimen CUMZ 1781 from Thepmuangthong Temple, Uthaithani (Clade C; Fig. 1B). **(E, F)** *Cyclophorus abditus* sp.nov. **(E)** holotype CUMZ 1828/1, and **(F)** paratype CUMZ 1828 from the type locality (Clade B; Fig. 1B).

**Figure 5:**
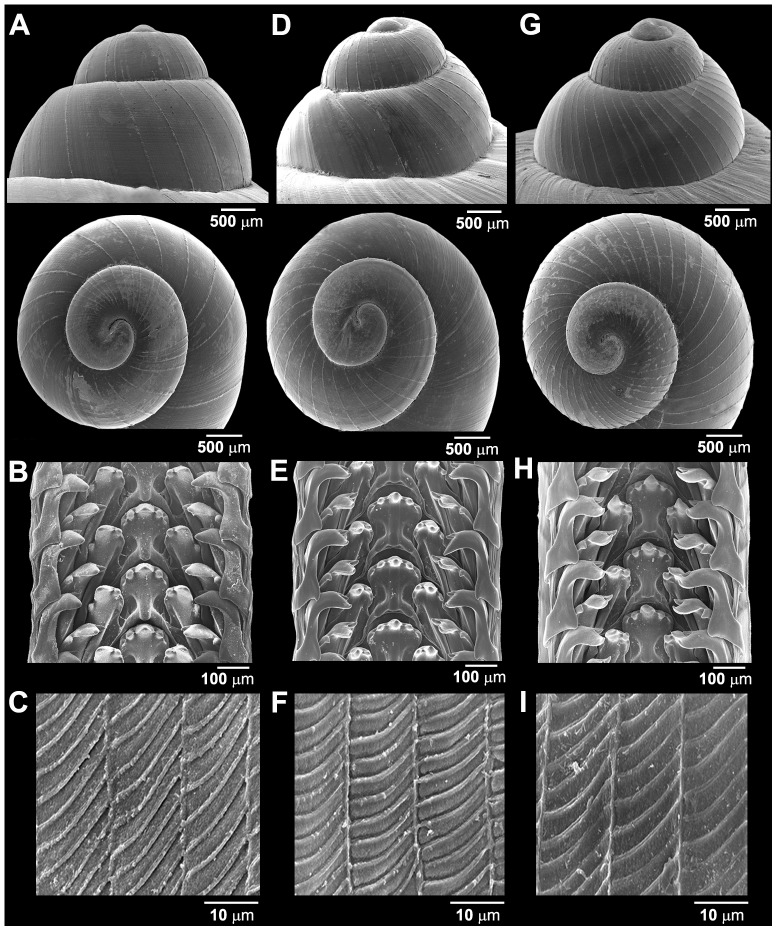
SEM pictures of (A, D, G) side and top views of protoconch, (B, E, H) radula morphology, and (C, F, I) surface sculpture of jaw of *Cyclophorus* species. **(A–C)** *Cyclophorus fulguratus* s.s. specimen CUMZ 1327 from Khao Chakan, Sra Kaeo (Clade A; Fig. 1B). **(D–F)** *Cyclophorus rangunensis* specimen CUMZ 1781 from Thepmuangthong Temple, Uthaithani (Clade C; Fig. 1B). **(G–I)** *Cyclophorus abditus* sp. nov. paratype CUMZ 1828 from the type locality (Clade B; Fig. 1B).

Most of the *C. fulguratus*–complex specimens examined in the present study showed a high similarity of shell shape, shell size, and reproductive morphology. However, we found that the three clades can be distinguished from each other by qualitative characters as follows: color pattern, pattern of protoconch and pattern of jaw (Figs. 4, 5).

## Discussion

### Molecular phylogeny

We have undertaken a detailed evaluation of species boundaries within the *C. fulguratus*– complex and examined its relationships with other congeners in the genus *Cyclophorus*. Our study included representative of 13 populations from previous studies [23], [26], [27] and 10 new additional populations. In general, the phylogenetic trees generated from analyses of individual genes (Figs. S1–S4) and from the concatenated datasets were congruent with all supporting the non-monophyly of the *C. fulguratus*–complex and the presence of three highly divergent *C. fulguratus*–complex clades: Clade A, the Eastern population; Clade B, the Northeastern population; and Clade C, the Central-West population. Some analyses also suggested the further division of Clade B into Upper and Lower Northeastern groups (separated by the congener *C. consociatus*) but this was not supported with all genes (Table S1).

Two lineages (Clade B, northeastern populations and Clade C, central-west populations; Fig. 1) were previously identified from karyotypic analysis [23], where it was suggested that *C. fulguratus* may represent two species, or one species undergoing speciation. A different karyotypic pattern was revealed with 12 m+2sm for central-west populations (Clade C in the present study) and 13 m+1sm for northeastern populations (Clade B in the present study). Later, the divergence of *C. fulguratus* into three lineages was supported by allozyme analysis based on 13 presumed allozyme loci of 10 enzyme systems [26]. The study suggested that the central, northeastern and eastern populations of *C. fulguratus* (Clade A, B, and C in the present study) have fixed or nearly fixed allelic differences at several allozyme loci. They observed relatively large divergences among these three regions and proposed an absence of gene flow between them. Moreover, a recent molecular phylogenetic study of the genus *Cyclophorus* based on COI, 16S rRNA, and 28S rRNA genes separated the *C. fulguratus*– complex into four groups: central (Clade C), eastern (Clade A), upper northeastern, and lower northeastern groups, with *C. consociatus* falling in between upper and lower northeastern groups [27]. Phylogenetic analysis in the present study did not resolve the Upper and Lower northeastern groups as two separate units (Clade B). Furthermore, the separation of Upper and Lower northeastern groups was not significantly supported in Bayesian species delimitation analysis. These findings suggest that the Upper and Lower Northeastern groups should be considered as a single evolutionary unit, though we note that our sampling of the Lower Northeastern group is at presented limited.

### Cryptic species within *C. fulguratus*

Although genetic and geometric-morphometrics of shell shape allowed for significant distinction of these three groups, there was a considerable degree of overlap of shell shape between groups. The genital system of this snail also shows high similarity [23], [24] and cannot be used to discriminate. Populations of the present species that are discontinuously distributed may be subject to reduced gene flow or a complete lack of genetic exchange. Over time, these populations may have allopatrically diverged into different species (Clades A, B and C) with little differentiation in shell morphology [53]. Alternatively, under similar selective pressure, their shell characters may have undergone convergent evolution [54].

These characters support the three clades as follows: (i) color pattern: scatter spot around shell in Clade C versus mostly chestnut brown shell in Clade B, and streaks and zigzags around the shell in the Eastern group (Clade A); (ii) pattern of protoconch: wide miniature line radial lamellae throughout the first 2 whorls of protoconch in Clade C versus miniature narrow radial lamellae throughout the first 2½ whorls of protoconch in Clade B and miniature narrow radial lamellae throughout the first 1½ whorl and then wide radial lamellae of the protoconch in Clade C; while (iii) pattern of jaw: round horizontal lines between block of jaw in Clade C, small groove running in the middle of horizontal line of jaw in Clade B and flat horizontal lines and shallow groove between blocks of jaw in Clade C.

The multiple lines of evidence presented in this study strongly suggest that the three geographical populations of the *C. fulguratus*–complex in Thailand should be recognized as three distinct species. Two of these are described species, *C. fulguratus* s.s. (Clade A) and *C. rangunensis* (Clade C), but the third (Clade B) is as yet undescribed and is described hereafter as *C. abditus* sp. nov.

### Systematic description

**Family Cyclophoridae Gray, 1847**

**Genus** ***Cyclophorus*** **Montfort, 1810**

***Cyclophorus fulguratus*** **(Pfeiffer, 1854)**

**Figs 4A-B** **,** **5A-C**

*Cyclostoma* (*Cyclophorus*) *fulguratum* Pfeiffer, 1854 [1852: 63]. Type locality: unknown [25]. Pfeiffer, 1854: 345, pl.45, fig. 9, 10 [55].

*Cyclophorus fulguratus*—Reeve, 1861: sp. 35 [6]. Kobelt, 1902: 112 [2]. Hanley and Theobald, 1876: 57, pl. 144, fig. 1 [56]. Kongim et al., 2006: 1-8 (in part) [23]. Prasankok et al., 2009: 900-906 (in part) [26]. Nantarat et al., 2014: 11, fig. 8a, b [28]

#### Material examined

Lectotype NHMUK 20130117/1 (Fig. 4A) and paralectotypes NHMUK 20130117/2-3 (2 shells) [28]. Plieu National Park, Chanthaburi: CUMZ 822 (3 shells), 863 (2 shells), 1180 (8 shells). Makok Waterfall, Chanthaburi: CUMZ 1135 (10 shell). Khao Soi Dao Waterfall, Chanthaburi: CUMZ 1076 (6 shells). Khao Sukim Temple, Chanthaburi: CUMZ 1224 (2 shells). Khao Chakan, Sra Kaeo: CUMZ 1327 (20 shells; Fig. 3B). Khao Maka Cave, Sra Kaeo: CUMZ 1688 (13 shells), 1788 (3 shells), 1821 (3 shells). Sapanhin Waterfall, Trat: CUMZ 1614 (6 shells).

#### Measurements

Shell height: ranges 24.7–30.6 mm and average 27.7±0.16 mm. Shell width: ranges 28.2-34.0 and average 31.1±0.16 mm.

#### Description

Shell medium, solid and globose turbinated. Spire elevated, apex acute; whorls 5 to 6 and convex; suture deep and wide. Periostracum thick to thin brownish corneous. Protoconch surface with thin distant transverse ridges (Fig. 5A). Following whorls with only irregular growth line. Last whorl rounded, and enlarge with white colour. Shell colour elegantly painted with angulated zigzag dark brown streaks on upper shell surface; on periphery with dark spiral band; lower periphery with striated stripe surrounded umbilicus. Aperture circular, slightly oblique; lip expanded and reflected with white to pale orange colour. Umbilicus rimate, wide and deep. Operculum corneous, multi-spiral and little concaved center (Fig. 4B)

#### Radula and jaw

Taenioglossan radula, each row contains 7 teeth with formula 2-1-1-1-2. Central tooth with well develop central cusp and two smaller lateral cusps on each side; central cusp largest with pointed tip; four lateral cusps on both sides perform triangular shape with pointed head. Lateral teeth consisted 2 cusps; outer cusp large, convex shape, and flanked with smaller inner lateral cusps. Inner marginal teeth composed of 3 cusps; central cusp large and convex head, and flanked with smaller and pointed head of one inner and one outer lateral cusps. Outer marginal teeth consisted 2 cusps; outer cusp large triangular shape with convex head, and flanked with smaller inner cusp with pointed head (Fig. 5B).

Jaw consists of two parts, each part thin and rhomboid shape. Sculpture with thin longitudinal parallel ridges, connected to each other with strong parallel slanting transverse ridges, which made sculpture of jaw like underside of leaf blade or block (Fig. 5C).

#### Distribution

The ranges of this species is demarcated to several localities in eastern Thailand, and probably in Cambodia and southern of Vietnam (Schileyko, 2011).

***Cyclophorus rangunensis*** **Kobelt, 1908**

**Figs 4C-D** **,** **5D-F**

*Cyclophorus fulguratus* var. Pfeiffer, 1869: 440, pl. 98, figs 1, 2. Type locality: Thyet-Mio et Rangoon in Burma, Pegu [57]. [Thayet District in Magway Region, Yangon and Bago, Myanmar].

Cyclophorus (Glossostylus) fulguratus var. rangunensis Kobelt, 1908: 647, pl. 93, figs 1, 2 [2].

*Cyclophorus fulguratus rangunensis* — Gude, 1921: 61 [1]. Nantarat et al., 2014: 21, fig. 17a, b [28].

*Cyclophorus fulguratus*— Kongim et al., 2006: 1-8 (in part) [23]. Prasankok et al., 2009: 900-906 (in part) [26].

#### Material examined

Lectotype NHMUK 20130091/1(Fig. 4C) and paralectotypes NHMUK 20130091/2-3 (2 shells) [28]. Thepsatit Temple, Nakhon Sawan: CUMZ 809 (20 shells). Khao Noh, Nakhon Sawan: CUMZ 1064 (5 shells), 1065 (4 shells). Pha Subin, Nakhon Sawan: CUMZ 1164 (5 shells). Klong Lan Waterfall, Kampaengphet: CUMZ 1602 (3 shells). Thepsathaporn Temple, Uthaithani: CUMZ 853 (3 shells), CUMZ 1232 (18 shells). Thepmuangthong Temple, Uthaithani: CUMZ 1781 (4 shells; Fig. 3D). Khao Nang Rum, Uthaithani: CUMZ 1061 (3 shells), 1062 (2 shells), 1063 (2 shells). Ramkamhaeng National Park, Sukhothai: CUMZ 839 (3 shells), 1188 (4 shells), 1189 (20 shells). Srisatchanarai Historical Park, Sukhothai: CUMZ 1201 (3 shells), 1427 (5 shells), 1430 (2 shells), 1476 (14 shells). Doi Haumod Mountain, Um-pang, Tak: CUMZ 1747 (8 shells). Bhumibol Dam, Sam Ngao, Tak: CUMZ 859 (5 shells), CUMZ 1176 (10 shells). Khao Bin Cave, Ratchaburi: CUMZ 1376 (3 shells). Tam Sue Temple, U-thong, Suphanburi: CUMZ 1401 (2 shells).

#### Measurements

Shell height: ranges 22.9–34.6 mm and average 28.9±0.33 mm. Shell width, ranges 26.4–38.4 and average 33.6±0.37 mm.

#### Description

Shell medium, solid and globose turbinated. Spire little depressed, apex acute; whorls 5 to 6, convex; suture wide and deep. Periostracum thick to thin brownish corneous. Protoconch with thin discernible transverse ridges (Fig. 5D). Following whorls with only irregular growth line. Last whorl rounded, enlarge and with white colour. Shell colour with fine brownish spiral lines and angulated brownish zigzag streaks like scatter spots on upper shell surface; on periphery with narrow dark spiral band; below periphery with pale brown broad spiral stripe surrounded umbilicus. Aperture circular, slightly oblique; lip expanded and reflected with white to pale orange colour. Umbilicus open and deep. Operculum corneous, multi-spiral and little concaved center (Fig. 4D)

#### Radula and jaw

Teeth shape and jaw sculpture are similar to that of *C. fulguratus*, only minor variation occur. Lateral and marginal teeth having pointed cusp (Fig. 5E), and jaw with strong longitudinal parallel ridges and parallel slanting transverse ridges (Fig. 5F).

#### Distribution

We have examined the specimens mentioned under the name *C. fulguratus* s.l. in Gude (1921: 62), which they show similar colour pattern to this species. Therefore, the distribution range of this species is from several localities in Burma (Kobelt, 1902; Gude, 1921), and included western to central of Thailand.

#### Remark

This species differ from *C. fulguratus* s.s in having chestnut brown streaks like scatter spots, relatively larger shell size, and the sculpture of jaw with deep grooves between blocks and horizontal line round.

***Cyclophorus abditus*** **Nantarat and Panha, sp. nov. urn:lsid:zoobank.org:act:EF38E373-EBBB-40D0-9B90-CAB760AB21E7**

**Figs 4E-F** **,** **5G-I**

*Cyclophorus fulguratus* —Kongim et al., 2006: 1-8 (part) [23]. Prasankok et al., 2009: 900-906 (part) [26].

#### Type materials

Holotype CUMZ 1828/1 (Fig. 4E; height 25.5 mm, width 29.8 mm, 5 whorls). Paratypes CUMZ 1828 (14 shells, Fig. 4F), 848 (2 shells), 849 (3 shells), 1165 (20 shells).

#### Type locality

Phu Kum Khao (Sirindhorn Museum), Sahat Sakhan, Kalasin ( =  locality no. 28 in Table 1; 103° 31′ 25.72″ E, 16° 41′ 44.67″ N; elevation 224 MSL), the sand stone mountains with the dry dipterocarp forest.

#### Etymology

The specific epithet is from the Latin word “*abditus*” meaning “hidden or concealed”. It refers to this mysterious new species has long been cryptic in the *C. fulguratus*. Description of this new species is here attributed to the first and the last author, Nantarat and Panha, respectively.

#### Other material examined

Nawa, Nakhon Phanom: CUMZ 812 (5 shells), 1158 (2 shells), 1399 (29 shells). Numphung Dam, Sakon Nakhon: CUMZ 851 (2 shells), 1145 (11 shells). Lumpahung, Sakon Nakhon: CUMZ 1750 (20 shells). Nanghong cave, Sakon Nakhon: CUMZ 1690 (12 shells). Waritchaphum, Sakon Nakhon: CUMZ 1838 (19 shells). Nam-un Dam, Sakon Nakhon: CUMZ 1839 (9 shells). Namlad, Sakon Nakhon: CUMZ 1840 (11 shells). Ban Dong Kum Pho, Sakon Nakhon: CUMZ 847 (1 shell), 1005 (3 shell). Phu Phan Cave, Sakon Nakhon: CUMZ 1609 (3 shells). Phu Pha Man, Nongbua Lamphu: CUMZ 1171 (3 shells). Tam Numthip Temple, Roi-et: CUMZ 1826 (3 shells). Road No. 212 from Nakhon Phanom to Dong Luang, Mukdahan: CUMZ 1841 (3 shells). Phu Khiao, Chaiyaphum: CUMZ 1254 (3 shells). Kang Lumduan Waterfall, Ubonratchathani: CUMZ 1827 (3 shells).

#### Measurements

Shell height: ranges 21.8–27.7 mm and average 24.6±0.16 mm. Shell width, ranges 25.8–32.0 and average 29.3±0.15 mm.

#### Diagnosis

This new species can be distinguished from *C. fulguratus* s.s. and *C. rangunensis* by having relatively smaller shell, uniform brown to brownish shell colour, protoconch with dense radial ridges, and lip little expanded. While, *C. fulguratus* performs relatively larger shell, with dark brown zigzag streaks and broader peripheral band. In addition, *C. rangunensis* exhibits brownish spiral lines and scatter spots on upper shell surface, and thicken expanded lip.

#### Description

Shell medium, solid and globose turbinated. Spire little depressed, apex acute; whorls 5 to 6, convex; suture deep and wide. Periostracum thick brownish corneous. Protoconch with thin closely discernible transverse ridges (Fig. 5G). Following whorls with only irregular growth line. Last whorl rounded, enlarge and with white colour. Shell colour usually with uniform brown to brownish colour, sometime dark zigzag streak present on spire; on periphery with narrow dark spiral band; lower periphery with narrow striated stripe surrounded umbilicus. Aperture circular, slightly oblique; lip little expanded and reflected with white colour. Umbilicus widely open and deep. Operculum corneous, multi-spiral and little concaved center (Fig. 4E, F)

#### Radula and jaw

Teeth shape and jaw sculpture are similar to that of *C. fulguratus*, only minor variation occur. Lateral and marginal teeth having pointed and curved central cusps (Fig. 5H), and jaw with strong longitudinal parallel ridges and parallel slanting transverse ridges (Fig. 5I).

#### Distribution

This new species is known from several localities in the northeastern Thailand. The application of the taxon name in previously publications by Kongim at al. (2006) [23] and Prasankok et al. (2009) [26] as the *C. fulguratus* s.l. are here reconsidered as the new species.

## Conclusions

*Cyclophorus fulguratus* s.l. is a highly variable species group that many malacologists [2], [3], [58]-[60] have suggested warrants taxonomic revision. Our findings have demonstrated that the current taxonomy of *C. fulguratus* s.l. is indeed inaccurate. Using multiple approaches, including molecular phylogeny, statistical tests of alternative hypotheses, Bayesian species delimitation, geometric morphometrics, and morphological data, we demonstrate that the species complex comprises three cryptic species, *C. fulguratus* s.s., *C. rangunensis*, and *C. abditus* sp.nov. in Thailand. Allopatric speciation may play an important role here, with mountain ranges and isolation by distance likely to be the main mechanisms driving the population structure of this snail. This study helps to clarify species limits in a genetically fragmented group and provides a framework for identifying and defining the cryptic lineage diversity of land snails. In the present day, fragmented habitats, fragile ecosystems, pollution, climate change and improper harvesting can cause decreasing population sizes [27]. Our species revisions will prove informative for informing conservation policies of these edible snails and protecting their habitat in Thailand.

## Supporting Information

Figure S1 Maximum-likelihood phylogenetic tree of the *Cyclophorus fulguratus* species complex and related species constructed using the 18S gene (431 bp).
(TIF)

Figure S2 Maximum-likelihood phylogenetic tree of the *Cyclophorus fulguratus* species complex and related species constructed using the 28S gene (585 bp).
(TIF)

Figure S3 Maximum-likelihood phylogenetic tree of the *Cyclophorus fulguratus* species complex and related species constructed using the 16S gene (470 bp).
(TIF)

Figure S4 Maximum-likelihood phylogenetic tree of the *Cyclophorus fulguratus* species complex and related species constructed using the COI gene (660 bp).
(TIF)

Table S1 Tree topologies of Clade B for each dataset.
(DOC)

Material S1 The concatenated dataset.
(FAS)
