# Supplementary figures and images for: Molecular Evidence for Cryptic Speciation in the Cyclophorus fulguratus (Pfeiffer, 1854) Species Complex (Caenogastropoda: Cyclophoridae) with Description of New Species
Source: PLoS One. 2014 Oct 9;9(10):e109785. doi: 10.1371/journal.pone.0109785 (PMC4192354; doi:10.1371/journal.pone.0109785)

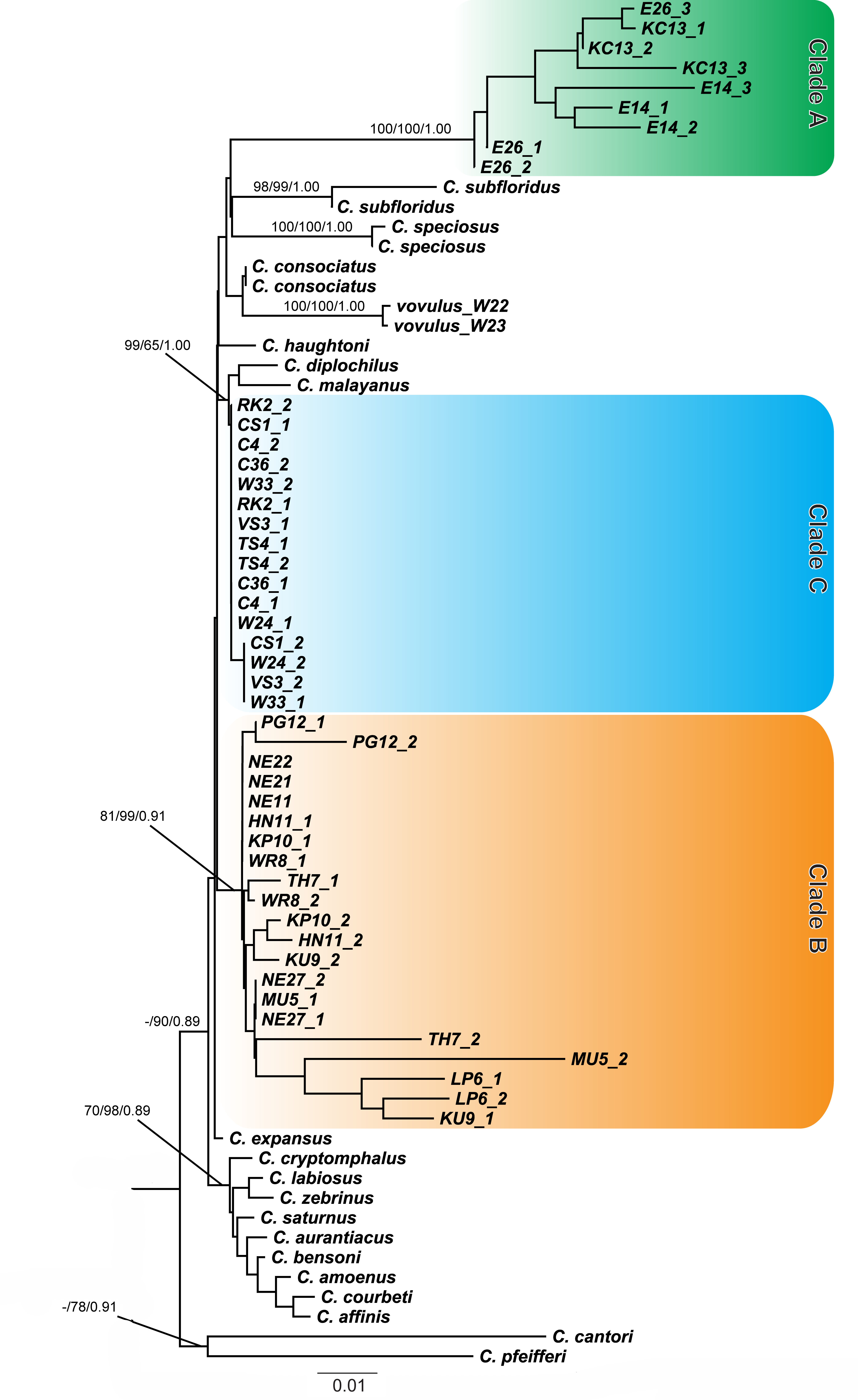

Supplement: Figure S1 — Maximum-likelihood phylogenetic tree of the Cyclophorus fulguratus species complex and related species constructed using the 18S gene (431 bp). (TIF) [file pone.0109785.s001.tif]

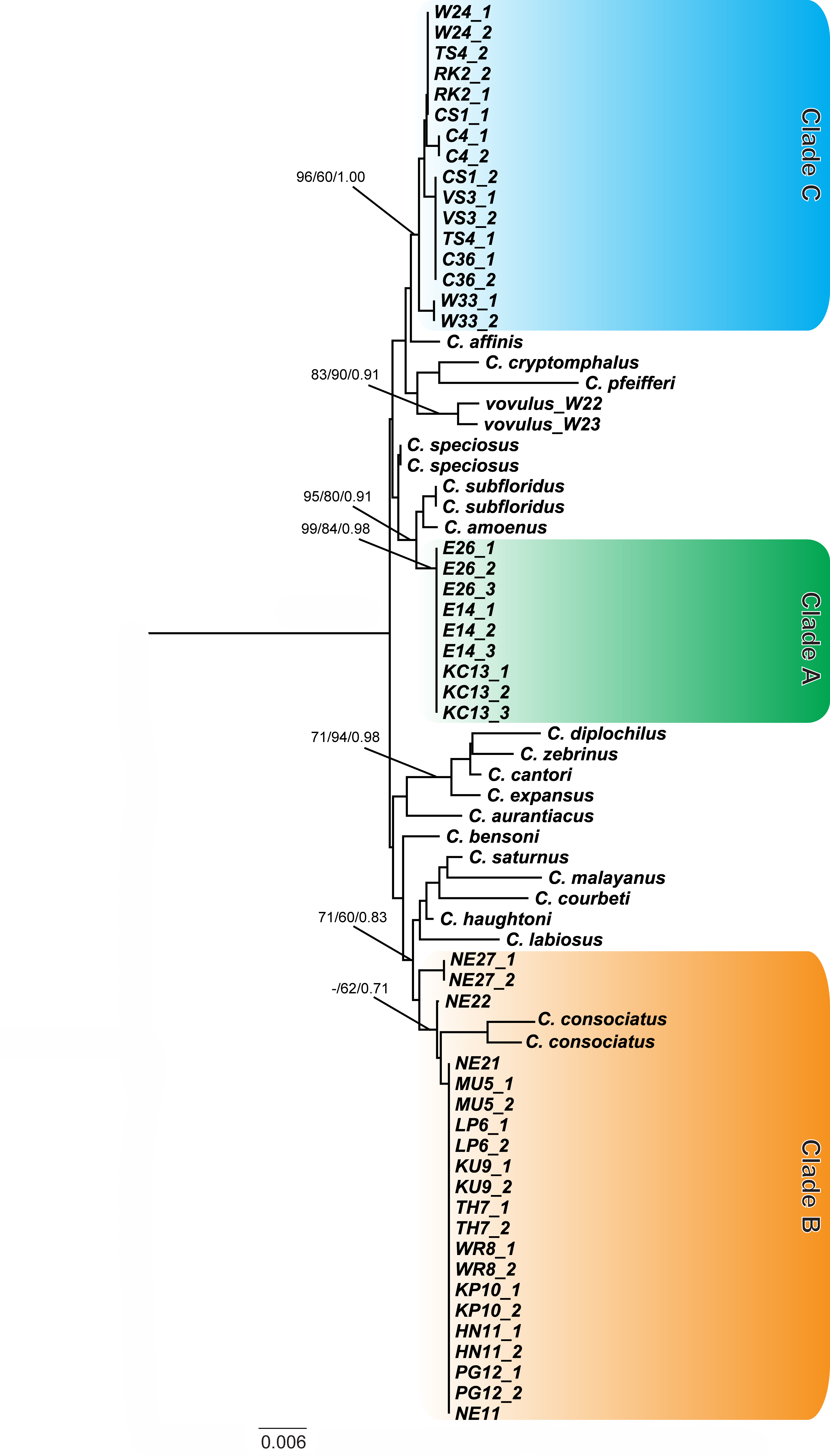

Supplement: Figure S2 — Maximum-likelihood phylogenetic tree of the Cyclophorus fulguratus species complex and related species constructed using the 28S gene (585 bp). (TIF) [file pone.0109785.s002.tif]

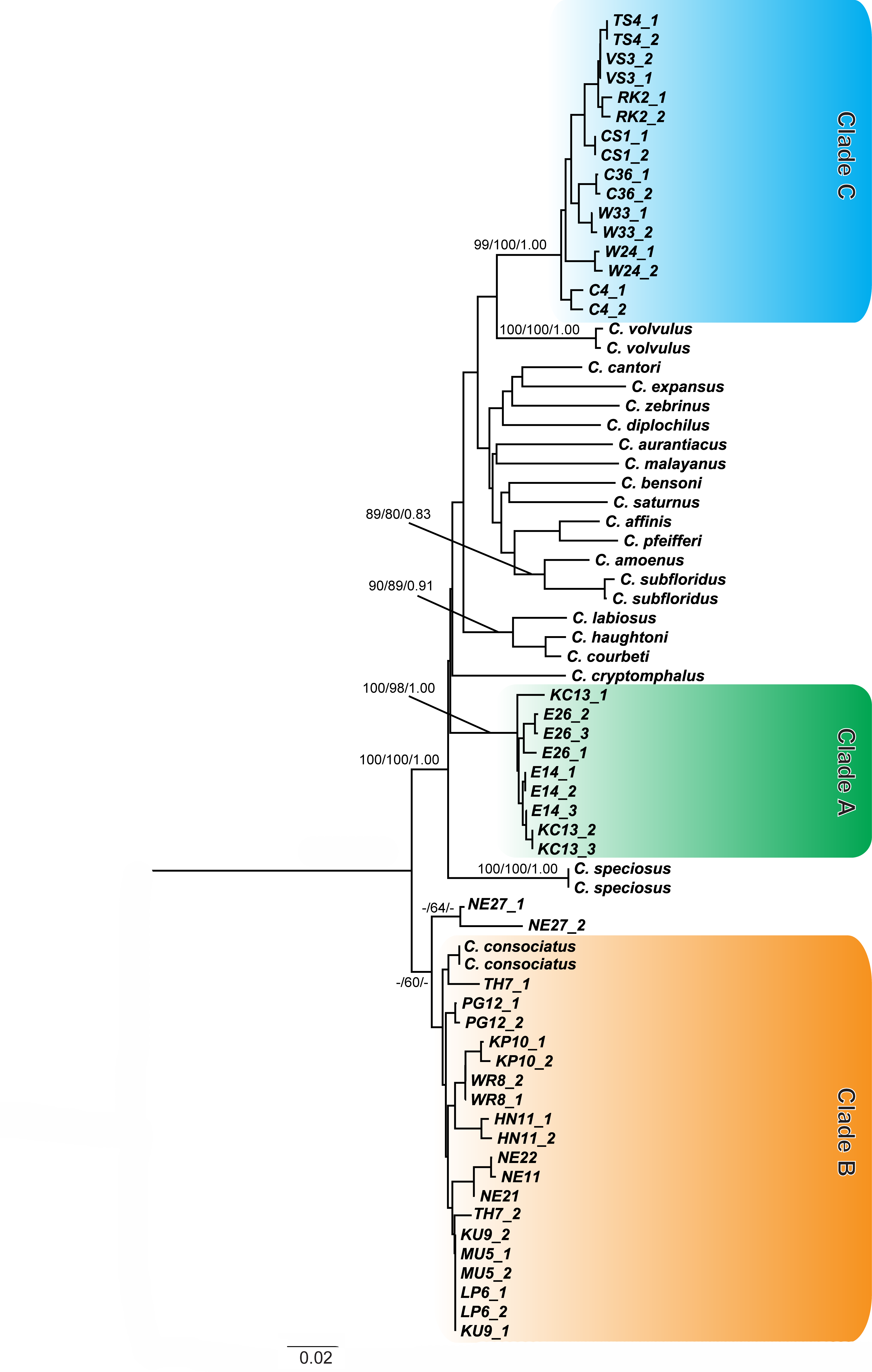

Supplement: Figure S3 — Maximum-likelihood phylogenetic tree of the Cyclophorus fulguratus species complex and related species constructed using the 16S gene (470 bp). (TIF) [file pone.0109785.s003.tif]

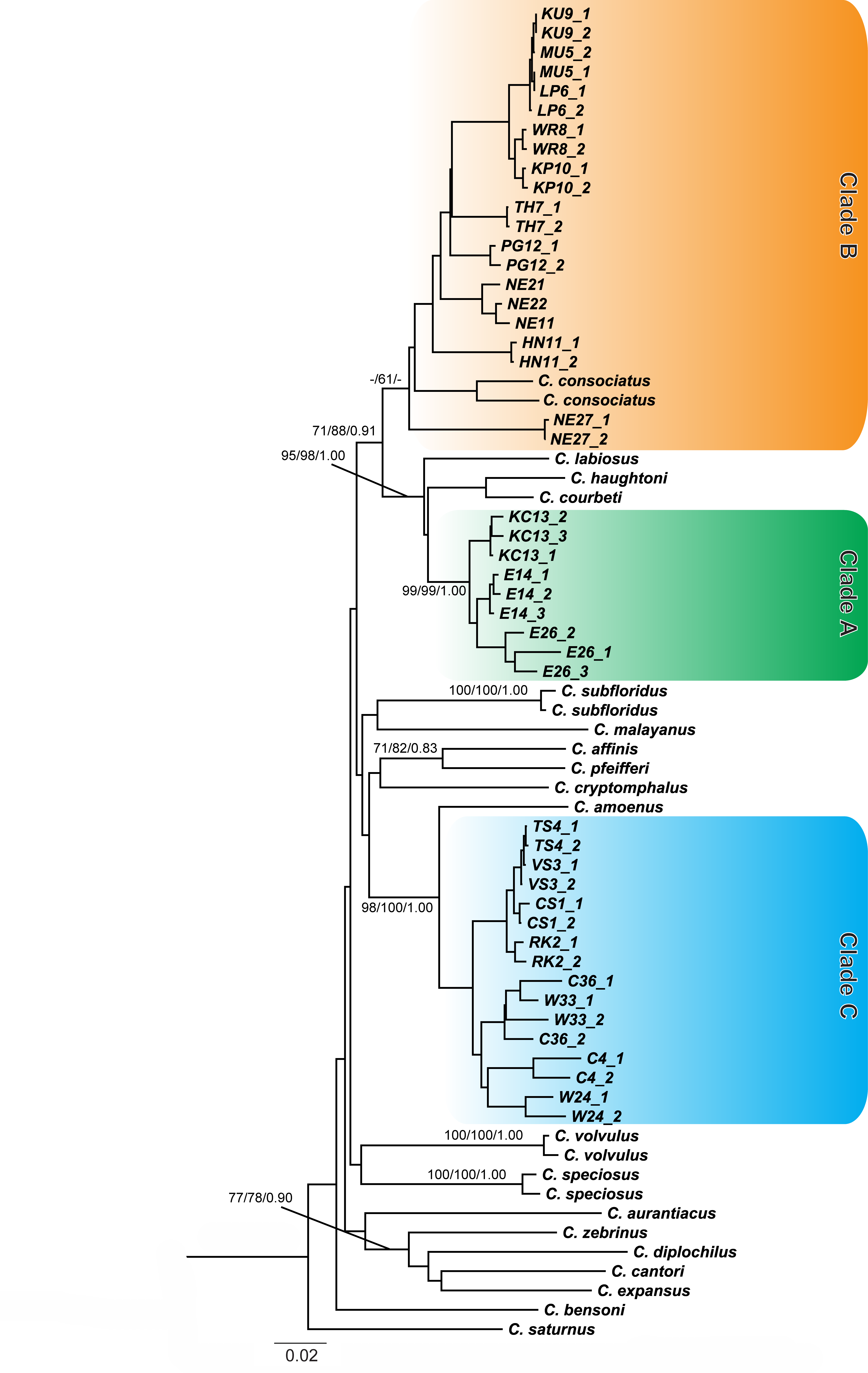

Supplement: Figure S4 — Maximum-likelihood phylogenetic tree of the Cyclophorus fulguratus species complex and related species constructed using the COI gene (660 bp). (TIF) [file pone.0109785.s004.tif]
