# Supplementary material for: Molecular Evidence for Cryptic Speciation in the Cyclophorus fulguratus (Pfeiffer, 1854) Species Complex (Caenogastropoda: Cyclophoridae) with Description of New Species
Source: PLoS One. 2014 Oct 9;9(10):e109785. doi: 10.1371/journal.pone.0109785 (PMC4192354; doi:10.1371/journal.pone.0109785)
Supplement: Table S1 — Tree topologies of Clade B for each dataset. (DOC) [file pone.0109785.s005.doc]

**Supplementary Table S1** Tree topologies of Clade B for each dataset**.**

Topology1: Clade B monophyly

Topology2: Clade B split into upper and lower northeastern and separated by *C. consociatus*

| **Datasets** | **Methods** | **Topology** |
| --- | --- | --- |
| 18S | NJ | Topology 1 |
|  | ML | Topology 1 |
|  | BI | Topology 1 |
| 28S | NJ | Topology 1 |
|  | ML | Topology 2 |
|  | BI | Topology 2 |
| 16S | NJ | Topology 1 |
|  | ML | Topology 2 |
|  | BI | Topology 1 |
| COI | NJ | Topology 1 |
|  | ML | Topology 2 |
|  | BI | Topology 2 |
| 16S, COI, 28S, 18S | NJ | Topology 1 |
|  | ML | Topology 1 |
|  | BI | Topology 1 |
